# Iron chelation by curcumin suppresses both curcumin-induced autophagy and cell death together with iron overload neoplastic transformation

**DOI:** 10.1038/s41420-019-0234-y

**Published:** 2019-12-09

**Authors:** Nathan E. Rainey, Aoula Moustapha, Ana Saric, Gael Nicolas, Franck Sureau, Patrice X. Petit

**Affiliations:** 1 CNRS UMR 8003, SSPIN Saints-Pères Neurosciences Institute, Paris University, Saint-Germain Campus, 45 rue des Saints-Pères, 75006 Paris, France; 2 INSERM U1148, Laboratory for Vascular Translational Science, UFR SMBH, Université Paris 13, Sorbonne Paris Cité, F-93017 Bobigny, France; 3 Division of Molecular Medicine, Ruder Boškovic Institute, Bijenička cesta 54, 10000 Zagreb, Croatia; 4 Medical faculty Bichat, UMR 1149—ERL CNRS 8252—Université Paris Diderot Paris 7, 16 rue Henri Hucherd, F-75890 Paris, France; 5 Laboratoire Jean Perrin, Université Pierre et Marie Curie—Paris 6, CNRS FRE 3231 Case Courrier 138, 4 place Jussieu, F-75252 Paris cedex 05, France; 6 Present Address: Head of the pharmacology and toxicology department, Faculty of pharmacy Albaath university, Homs, Syria

**Keywords:** Mitophagy, Cell biology, Cell death

## Abstract

Iron overload, notably caused by hereditary hemochromatosis, is an excess storage of iron in various organs that causes tissue damage and may promote tumorigenesis. To manage that disorder, free iron depletion can be induced by iron chelators like deferoxamine that are of increasing interest also in the cancer field since iron stock could be a potent target for managing tumorigenesis. Curcumin, a well-known active substance extracted from the turmeric rhizome, destabilizes endoplasmic reticulum, and secondarily lysosomes, thereby increasing mitophagy/autophagy and subsequent apoptosis. Recent findings show that cells treated with curcumin also exhibit a decrease in ferritin, which is consistent with its chemical structure and iron chelating activity. Here we investigated how curcumin influences the intracellular effects of iron overload via Fe-nitriloacetic acid or ferric ammonium citrate loading in Huh-7 cells and explored the consequences in terms of antioxidant activity, autophagy, and apoptotic signal transduction. In experiments with T51B and RL-34 epithelial cells, we have found evidence that curcumin-iron complexation abolishes both curcumin-induced autophagy and apoptosis, together with the tumorigenic action of iron overload.

## Introduction

Iron is a key element of numerous biological processes, but the presence of free or loosely bound iron can be toxic to cells^1^. Iron being an active redox metal, the excess free form can generate reactive oxygen species (ROS) through Haber–Weiss reduction followed by Fenton reactions^2,3^. The liver plays a major role in regulating iron storage in the case of excess iron or iron uptake in the case of deficiency. Aberrant iron accumulation can lead to cirrhosis and hepatocarcinoma, but also to heart damage, joint, and metabolic disorders^4,5^. Phlebotomy remains the mainstay of iron-overload diseases, but iron chelation therapy has proven to be a valuable alternative^6^.

Although iron chelation therapy was initially designed to alleviate the toxic effects of excess iron occurring in iron-overload diseases, the novel toxicological properties of some iron chelator complexes have radically shifted their intended applications toward cancer chemotherapy^7–9^. Some iron chelators are able not only to chelate iron, but also to inhibit the redox properties of free labile iron. Such ligands may prevent iron from participating in Fenton reactions, inhibiting the formation of ROS like the hydroxyl radical, which initiates oxidative damage^10^ and also ferroptosis, recently recognized as a form of regulated necrotic cell death^11^. Ferroptosis is usually designed as one of the multiple variants of cell death that can be characterized by a high intracellular level of free iron associated with ROS^12,13^. Ferroptosis is linked to the production of reduced glutathione and/or to alterations of glutathione peroxidase 4 (GPX4), which usually acts as a ROS controller^14–16^. Additive markers of ferroptosis are lipid peroxidation and protein carbonylation leading to more ROS and death signaling^10,17^. Interestingly, some genetic disorders of iron metabolism and/or chronic inflammation often progress to iron overload and recent evidence shows, for example, the possibility of lowering brain iron accumulation with the membrane permeant chelator deferiprone in Friedreich ataxia^18–21^.

However, the choice of iron chelators is critical. Normally, the chelation of excess iron gives rise to a more inactive complex, hence providing a useful method of preventing the toxic effects of iron-overload diseases. In contrast, some chelators enhance the production of ROS after complexation with Fe^2+^. These chelators may induce Fe^2+^ toxicity of interest in cancer chemotherapy^22,23^. By rapidly depleting proliferating cancer cells of Fe^2+^, iron chelators can also inhibit the activity of Fe that acts as coenzyme to essential steps of DNA synthesis^24–26^. In addition, Fe^2+^ depletion is known to affect the expression of molecules involved in cell cycle progression and growth, i.e. N-myc downstream regulated gene 1, cyclin D1, cyclin A and p21^waf1^ leading to G1/S arrest in the cell cycle^27–29^. These effects combined with ROS generation provide multiple mechanisms of action mediated by Fe^2+^ chelation to inhibit tumor growth.

Curcumin is the major chemical component of turmeric, a dietary spice made from the root of *Curcuma longa L*., which is used extensively in traditional Indian medicine^30^. Curcumin is a potent bioactive compound that is the subject of active study in the fields of cancer^31–33^, atherosclerosis^34^, steatohepatitis^35^, and neurodegenerative diseases, such as Alzheimer’s disease^36,37^ and Parkinson’s disease^38,39^, as well as for the promotion of wound healing^40–42^.

At the cellular level, curcumin's mechanisms of action are complex and multifactorial. We and others have previously addressed its mode of action and confirmed its hormetic nature^43–47^. At low concentration, curcumin has a very effective antioxidant activity, whereas at higher concentration (≥20 μM) it behaves as a potent pro-oxidant^47^.

In the field of cancer, curcumin inhibits the proliferation of tumor cells in vitro and in vivo. It also inhibits cell invasion, arrests cancer cells at the G2/M phase of the cell cycle, and induces autophagy. Furthermore, curcumin suppresses the activation of AKT, mTOR and P70S6K proteins^46,48,49^. Curcumin, therefore, is a potent tumor suppressor and simultaneously an inducer of autophagy. In the case of failure, this sequence of events leads to apoptosis^44^. Taken together, these data imply a fail-safe mechanism regulated by autophagy in the action of curcumin, suggesting a therapeutic potential for curcumin, which seems to offer a novel and effective strategy for the treatment of malignant cells^47^.

Curcumin strikingly modulates proteins of iron metabolism in cells and in tissues, suggesting that it has the properties of an iron chelator^50–53^. Because of its polyphenol structure, curcumin forms complexes with a number of different metal ions^54^ and especially with iron^51^. The α,β unsaturated β-diketone moiety of curcumin can form chelates with transition metals. Metal chelates of curcumin are mostly non-fluorescent, although absorption spectra show significant changes that can be tracked to assess the efficiency of chelation. Metal chelates of curcumin of types 1:1 and 1:2 have been reported for the ions Cu^2+^, Fe^2+^, Mn^2+^, Pb^3+^, and others^55^. All these metals can play a role in amyloid aggregation, and curcumin is being investigated as a chelating agent for use in the treatment of Alzheimer pathogenesis^54^. Since curcumin is lipophilic and readily crosses membranes, it may also chelate more metal ions intracellularly.^56^ Evidence shows that curcumin is more efficient when low amounts of iron are present intracellularly^53^. However, how curcumin binds to these metals has not been investigated in detail and nor have the consequences of this interaction in terms of autophagy and cell death mechanisms.

Here we demonstrate that the intracellular chelating activity of curcumin does not change the amount of iron loaded, but that its chelating activity is crucial in inhibiting iron overload cytotoxicity and further effects on liver cell lines. These findings position curcumin as a powerful alternative candidate for the control of liver damage linked to intracellular iron overload.

Moreover, we tested the capability of curcumin as a powerful iron chelator to prevent tumor promotion in T51B and RL-34 cells. Together with the fact that curcumin binds to iron and prevents iron toxicity^57–59^, we have found that curcumin chelation acts like deferoxamine^29^ by inhibiting tumor promotion.

In this context, curcumin may be effective in preventing chemically induced liver damage, by decreasing liver toxicity in rats in the case of galactosamine or carbon tetrachloride poisoning^60^, and also abolishes the carcinogenic effects of aflatoxin and/or nitrosodiethylamine^61,62^.

## Results

### Curcumin binds iron

**Fig. 1:**
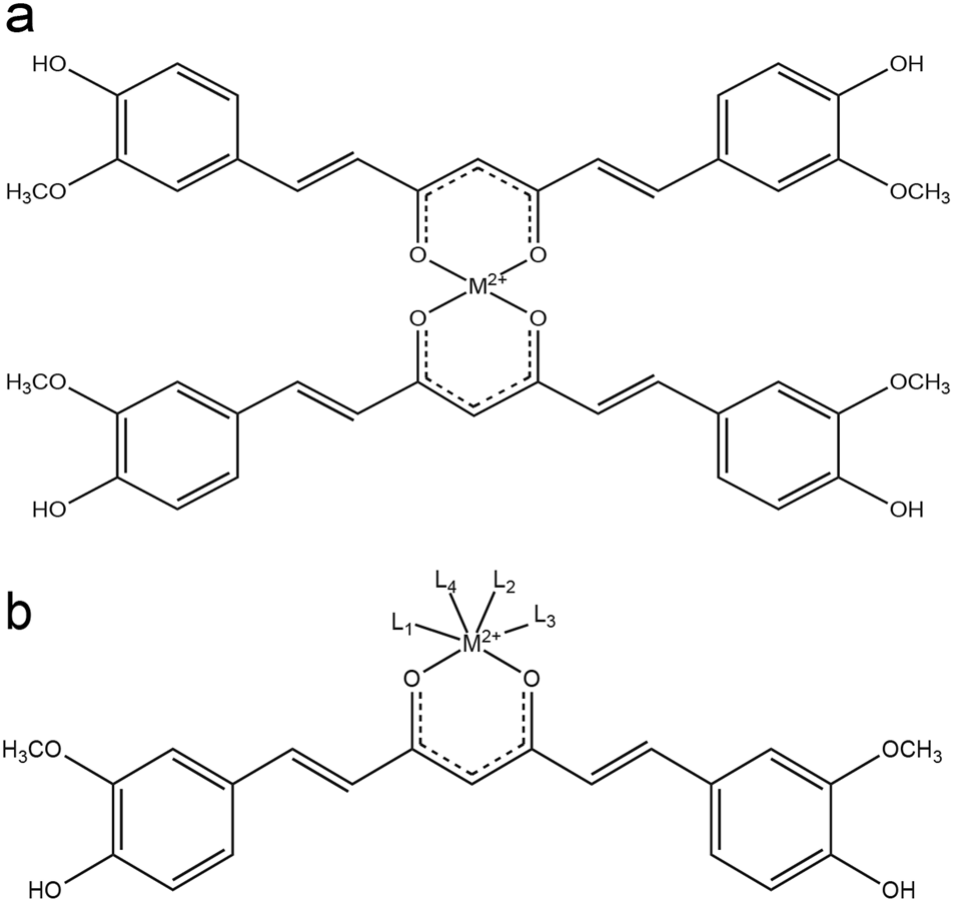
Curcumin chelates. Schematic representation of the curcumin chelation of bivalent metals. **a** Curcumin chelates can adopt a 2:1 configuration. **b** Curcumin can also chelate metals in conjunction with other ligands.

**Fig. 2:**
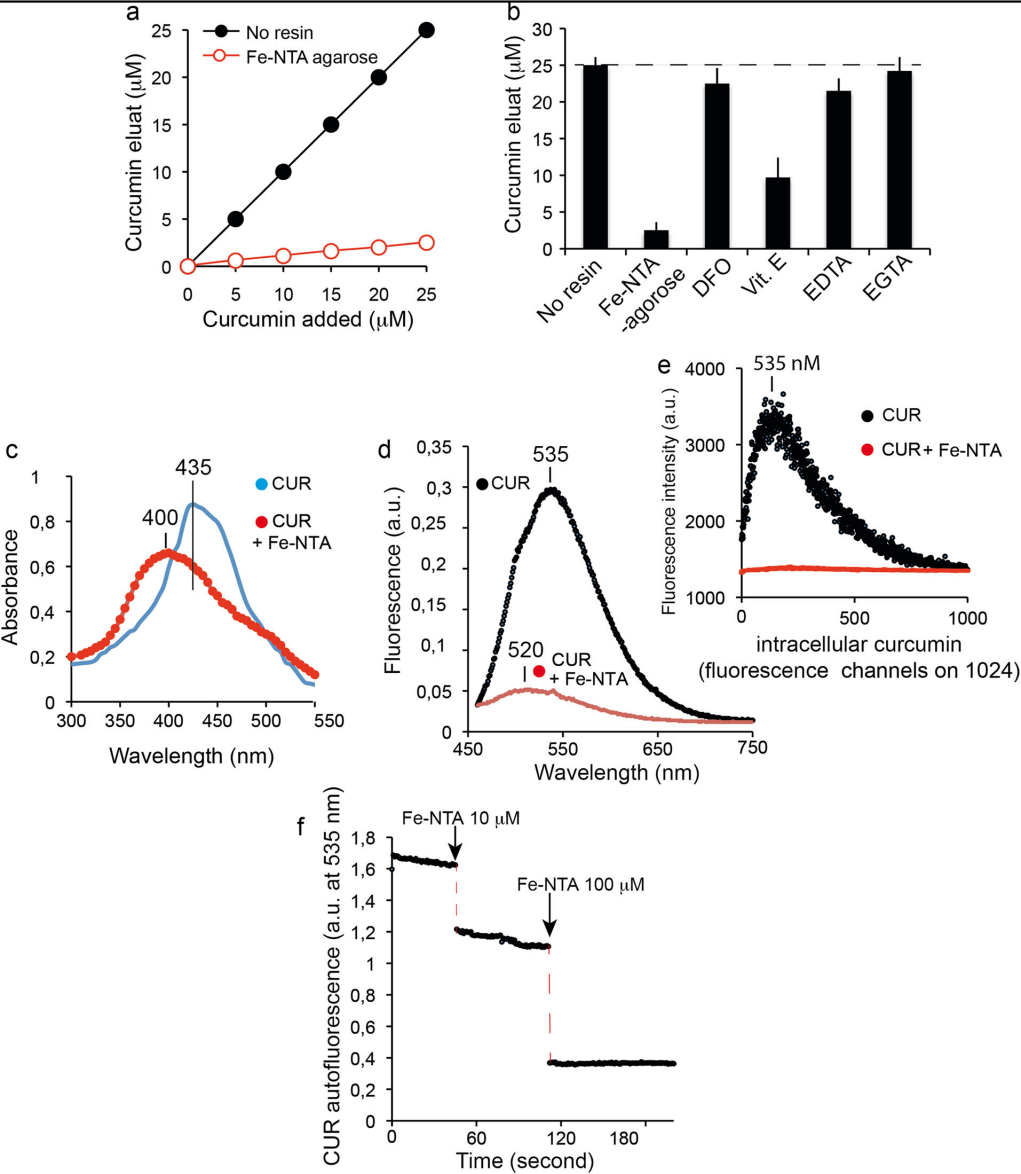
Curcumin binds iron. **a** Test of the removal of curcumin from solution by Fe-NTA resin. Increasing amounts of curcumin were incubated with Fe-NTA-agarose (black circles) or without (open circles). The free curcumin remaining in solution after removal of the resin was determined by absorbance at 435 nm against a calibration range. The experiments have been repeated more 12 times and the eman values are given in ±SD (but are so small that they are not vosibles on the figure drawing). **b** Inhibition of curcumin binding to iron. Curcumin (20 μM) was incubated alone (no NTA resin), with iron resin (Fe-NTA agarose), or with iron resin preincubated with excess deferoxamine (250 μM), excess vitamin E (1 mM), excess EGTA (2 mM) or excess EDTA (2 mM). Unbound curcumin was determined as for **a**. The means ± S.E. are presented from at least five independent determinations. **c** Absorbance spectrum of Huh-7 cells treated with 20 μM curcumin for 6 h with (in red) or without (in blue) 100 μM Fe-NTA loading for 24 h. **d** Fluorescence emission spectra of Huh-7 cells treated with 20 μM curcumin for 6 h (same conditions as in **c**). **e** Microspectrofluorimetry of Huh-7 cells treated with 20 μM curcumin for 6 h with recording of the curcumin emission fluorescence (same conditions as in **d**). **f** Effect of Fe-NTA (10 and 100 μM addition) on the fluorescence recording of a curcumin solution (20 μM) at 535 nm in a cuvette with excitation at 435 nm.

Spectroscopic shift techniques have shown that iron binds to curcumin in solution^54,63^. Figure 1 illustrates the capability of curcumin to chelate divalent cations, i.e., iron (Fe^2+^). We used an alternative metal affinity chromatography method with handmade Fe-NTA columns. Figure 2a shows the loading efficiency of curcumin from solution to the Fe-NTA-agarose column. Curcumin binding was rapid and efficient, reaching 90–95% after incubation for 10 min on the iron-containing resin. It is well known that the diketone groups of curcumin likely interact with the Fe-NTA-resin in a similar fashion to the phosphate oxygens of phosphopeptides, for which this iron affinity chromatography resin was developed^64^. The data extracted from Fig. 2 gave an affinity estimation around μM, in agreement with the high-affinity iron binding site of curcumin reported by Baum et al.^54^. Comparable curcumin binding was not observed when Fe was replaced by Ni as the metal ion^64^. A significant fraction of curcumin (based on the absorbance at 435 nm) was recovered from Fe-NTA-agarose upon addition of competitive iron chelators like deferoxamine (DFO) or EDTA (and EGTA to a lesser extent) (Fig. 2b). Vitamin E was a much less efficient iron chelator (Fig. 2b). These data reinforce previous reports of iron binding to curcumin and suggest that iron chelation may eventually enable curcumin to reduce both the uptake and toxicity of iron within cells.

The curcumin absorbance spectrum is modified by the presence of Fe-NTA: the absorbance of curcumin was shifted from 435 nm to 400 nm for the complex when bound to iron (Fig. 2c). The natural fluorescence of free curcumin, which peaks at 535 nm, was completely quenched when incubated with Fe-NTA, either in emission spectra or microspectrofluorimetry on cells (Fig. 2d, e). Acellular experiments also demonstrated that Fe-NTA added to curcumin induced instantaneous, dose-dependent quenching of curcumin fluorescence (Fig. 2f).

### Curcumin efficiently chelates Fe^2+^ but does not block iron uptake in Huh-7 cells

**Fig. 3:**
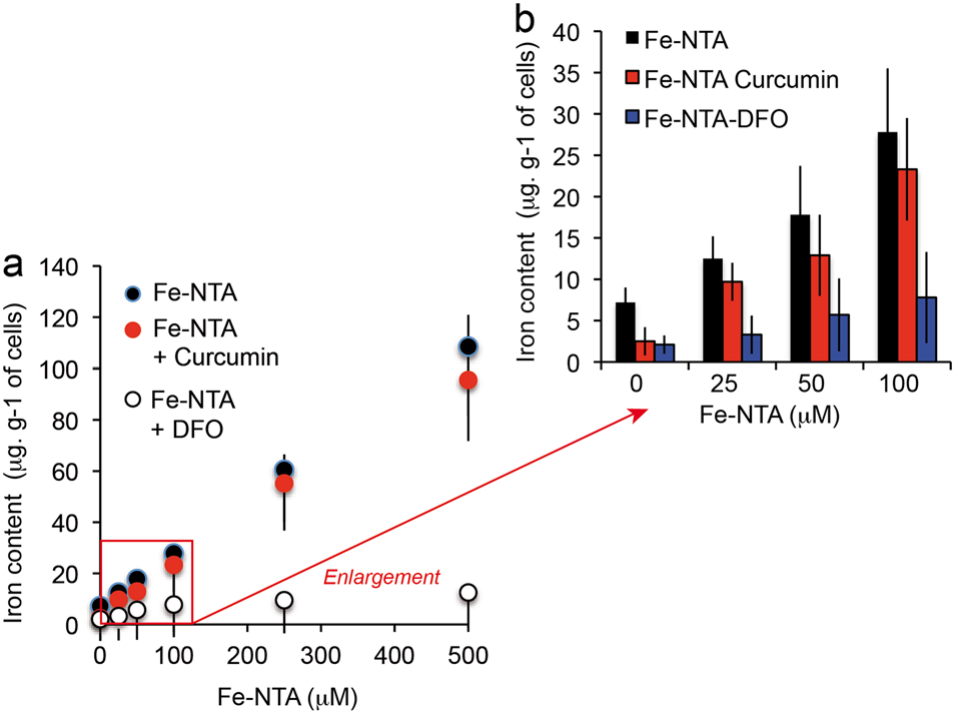
Cellular iron content after loading with Fe-NTA. **a** Fe-NTA cell loading (0–500 μM) and measurements of the intracellular iron content in presence of deferoxamine or curcumin (20 μM). **b** Enlargement of the Fe-NTA cell loading in the lower range between 0 and 100 μM in the presence or absence of deferoxamine (excess, 250 μM) or curcumin (20 μM). For a and b, the means ± S.E. are presented from at least 10 independent determinations.

The assay of intracellular iron showed that iron chelation by curcumin does not alter cellular iron uptake, whereas curcumin only slightly affected the total amount of intracellular iron (Fig. 3a, b) (the cells were loaded with iron before addition of curcumin to the medium). The detected cellular iron was in the range of 28 ± 10 μg/g of cells for 48-h incubation with 100 μM Fe-NTA, whereas control cells contained 7.2 ± 1.9 μg iron/g of cells (Fig. 3b). Pre-incubation with DFO and CoCl_2_ almost totally abolished the iron content of the cells treated with 100 µM Fe-NTA (Fig. 3b), meaning that DFO and CoCl_2_ act mostly on iron transport activity whereas curcumin acts intracellularly with an almost strict chelating activity (immobilization of the free intracellularly available Fe^2+^).

**Fig. 4:**
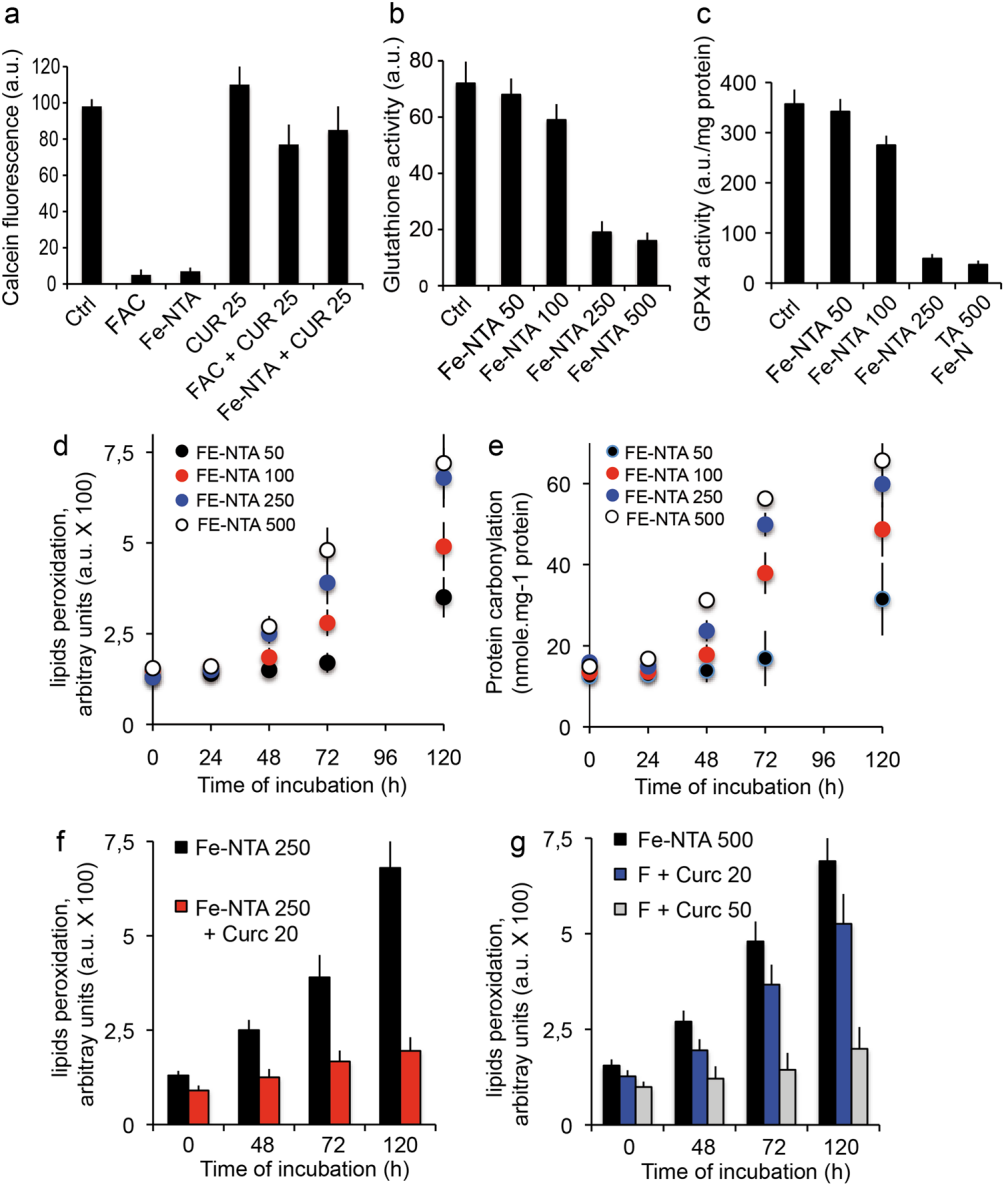
Cellular metabolic events. **a** Cells were charged with calcein-AM and either FAC (200 μM) or Fe-NTA (100 μM) and compared with cells loaded with curcumin only (20 μM). Cells were simultaneously treated with FAC and curcumin or Fe-NTA and curcumin. After 1 h, the cells were rinsed with phosphate buffer saline (PBS) on ice and calcein fluorescence was measured. (±SD for six repeated experiments). **b** Effect of iron loading by cell loading with 0–500 μM Fe-NTA and its effect on glutathione activity.(±SD for eight repeated experiments). **c** Effect of iron loading (as in **d**) on lipid peroxidation as a function of time (0–120 h). **d** Effect of iron loading (as in **d**) on protein carbonylation as a function of time (0–120 h). **e** Measurement of high doses of iron loaded with 250 µM Fe-NTA in the presence of 20 µM curcumin. **f** Measurement of high doses of iron loaded with 500 µM Fe-NTA and 20 µM or 50 µM curcumin. All experiments presented in c to f are the mean ± SD for 10 repeated experiments.

Additional experiments with calcein, whose fluorescence quenching is strictly correlated with intracellular accumulation of iron, demonstrated efficient quenching by Fe-NTA treatment and FAC. Also, whatever the treatment method used, curcumin complexation with iron completely restored calcein fluorescence (Fig. 4a).

### Toxicity features associated with Fe-NTA loading

Increased loading of Fe-NTA (from 50 to 500 μM) is linearly related to the intracellular iron content (Fig. 3a and Table 1) and is associated with a significant reduction of glutathione activity (Fig. 4b) and glutathione peroxidase activity (GPX4) (Fig. 4c). But there are significant changes only at high doses (≥100 μM). These events are associated with lipid peroxidation (Fig. 4d) and protein carbonylation (Fig. 4e). These characteristics are widely accepted as the main markers of ferroptosis^11,12,65^ that we discussed in the introduction. However, at the concentration of 100 μM Fe-NTA for 48 h that we currently use in our work, all four of these parameters are discrete and ferroptosis is already engaged in terms of metabolism, but there is not much detectable typical cell death.

**Table 1:** Regulation of the main metabolic events linked to ferroptosis induction.

Treatment	Control cells	+ Fe-NTA 500 µM	+ Fe-NTA 500 µM +Curcumin 20 µM	+ Fe-NTA 500 µM +Curcumin 50 µM
Iron amount µg/g cells	7.2 ± 1,9	114 ± 10.6	109 ± 9.5	109 ± 19.5
Gluthatione activity (a.u.)	74 ± 8,2	17 ± 5.3	39 ± 8.4	69 ± 7.9
GPX4 activity a.u./mg protein	370 ± 25.2	28 ± 7.2	130 ± 31.9	350 ± 29.4
Lipid peroxidation a.u. X 100	1.6 ± 0.3	7,3 ± 1.4	3,9 ± 0,9	1,7 ± 0.2
Protein carbonylation nmole/mg protein	17.3 ± 0.5	67,5 ± 8,7	29,9 ± 7.2	17,1 ± 0.4
Necroptosis (% of cells PI +++)	9 ± 3.2 Necrosis in culture	90 ± 6.4	39 ± 7.3	14 ± 5.9

### Fe-NTA loading of Huh-7 cells leads to ferroptosis

**Fig. 5:**
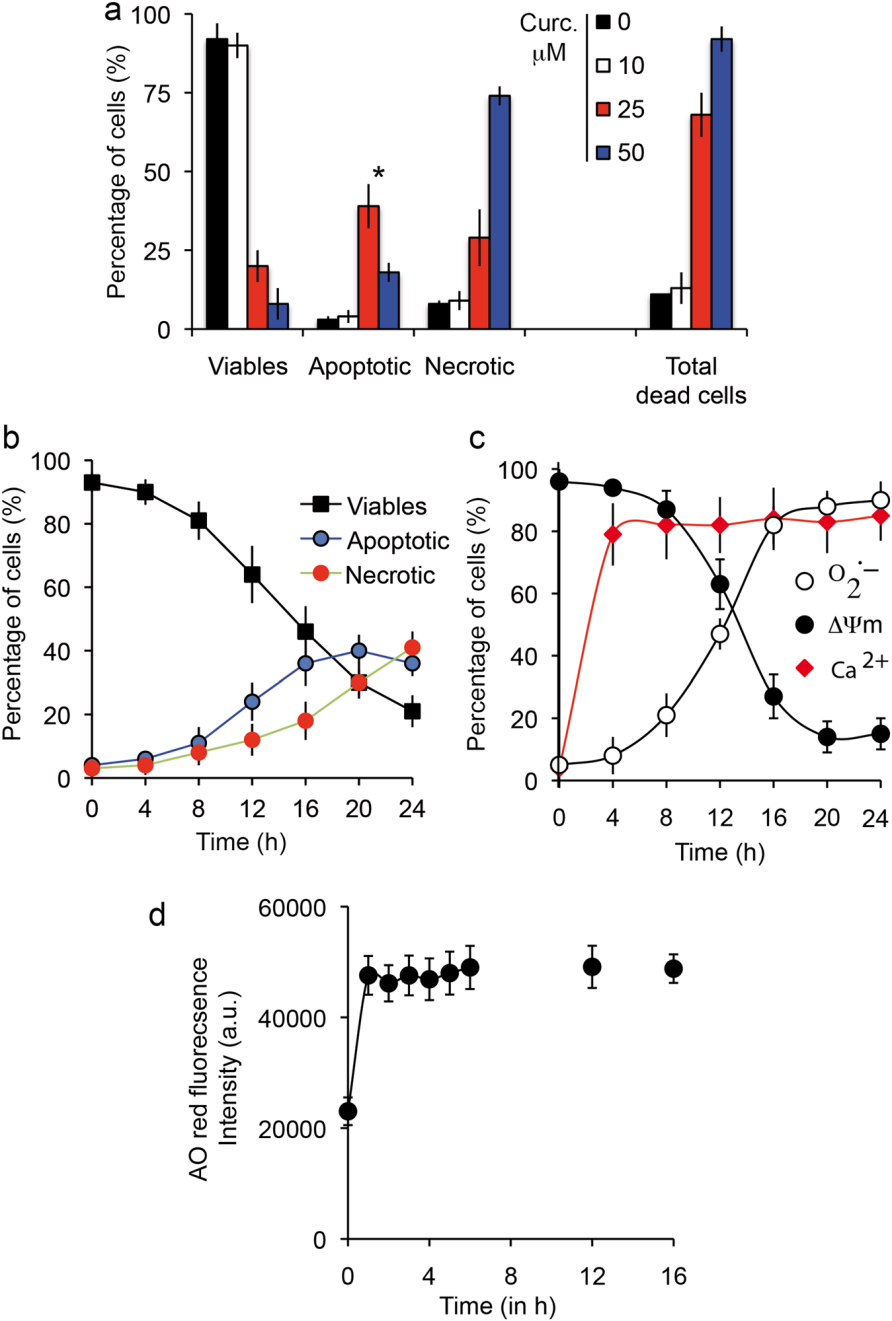
Prototypic induction of cell death by curcumin. **a** Flow cytometry analysis of Huh-7 cell death. Percentage of viable, apoptotic and necrotic cells among cells treated with various concentrations of curcumin (0–50 μM for 24-h incubation). YO-PRO-1/PI staining was used to analyze membrane permeability and discriminate three populations: YO-PRO-1^**−**^/PI^**−**^ for viable cells, YO-PRO-1^+^/PI^**−**^ or YO-PRO-1^+^/PI intermediate for apoptotic cells and YO-PRO-1^+^/PI^+^ for necrotic cells. Asterisk inidicates the maximal number of apoptotic cells was observed at 25 μM curcumin for 24 h. Data are expressed as the mean ± S.D. (*n* = 7). **b** Same as in panel a but plotted as a function of time from 0 to 24 h for 25 µM curcumin. Data are expressed as the mean ± S.D. (*n* = 7). **c** Metabolic measurements linked to dying cells after 24-h incubation with 25 µM curcumin. Mitochondrial membrane potential with DiOC_6_(3) fluorescence (ΔΨm, black circles), superoxide anion generation tested with MitoSOX-green fluorescence (O_2_^.−^, empty circles) and calcium increase measured with Fluo 4-AM (intracellular calcium, red diamonds). Data are expressed as the mean ± S.D. (*n* = 10).

**Fig. 6:**
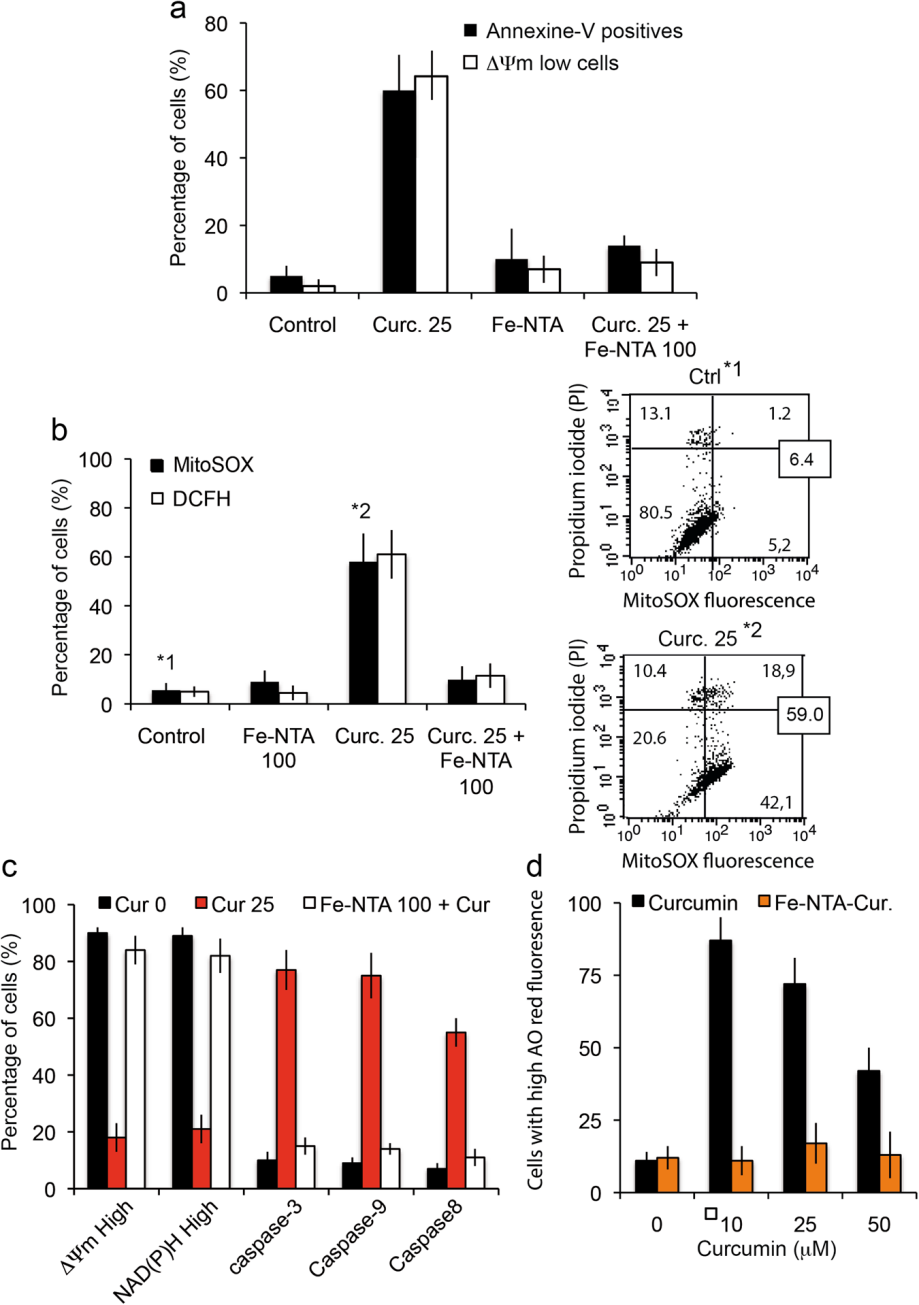
Cell death induced by curcumin and Fe-NTA-curcumin complex. **a** Flow cytometry analysis of early and late events during cell death (respectively, drop in mitochondrial membrane potential (ΔΨm) and PS exposure) when cells are treated for 24 h with curcumin (20 μM) or Fe-NTA (100 μM) alone or with both, which behave intracellularly as chelators. The results are issued from 7 independent experiments. **b** Flow cytometry analysis of the events following the ΔΨm drop (in a): generation of superoxide anions (MitoSOX staining) and hydroperoxide production (DCFH-DA staining. Each histogram is from seven independent experiments and ±SD is given. Control and 20 µM curcumin (24 h) conditions are illustrated respectively in 1* and 2* by biparametric plots together with the viability measurement (PI staining). The percentage of cells in each panel is given as a percentage (%) of the whole population. **c** Broad metabolic analysis showing that treatment with both products restores control conditions, with respectively a large population of high ΔΨm and NAD(P)H together with low activation of the so-called “initiation caspase” (caspase-8) and “executioner caspases” (caspase-3, -7 and caspase-9). Cells were treated with 20 µM curcumin or 100 µM Fe-NTA or both. **d** Cells were treated for 24 h with curcumin or Fe-NTA, or both. Then we analyzed red acridine orange fluorescence. As curcumin alone induces low pH vesicle staining which can be related to autophagy induction (more lysosomes and appearance of autophagic vesicles,) the Fe-NTA-curcumin complex abolishes that increase and returns to baseline level.

The effects of iron loading were assayed by multiparametric flow cytometric measurements, which indicate impaired cellular viability and also mitochondrial membrane potential drop (ΔΨm) and annexin-V positivity (Fig. 6a) and the generation of reactive oxygen species (Fig. 5b), whatever the H_2_O_2_ or superoxide anions generated at the mitochondrial membrane. Classic flow cytometry tests used to determine the induction and nature of cell death by Fe^2+^ overload show that this cell death is characteristic of apoptosis at very low concentrations [up to 100 μM Fe-NTA] and of typical ferroptosis at higher concentrations (>100 μM Fe-NTA, where free or loosely bound iron becomes more cytotoxic). If tested with annexin-V/PI staining, the final death process resembles that of necrotic cells (they are Annexin-V^+^ and PI^+++^). Iron-induced cell death is associated with a continuous loss of cell viability (plasma membrane permeability altered with first YO-PRO-1^+^ staining at PI^-^ and when [Fe^2+^] increases, the cells become YO-PRO-1^+^/PI^+^, as do necrotic cells. But some characteristics of Fe-NTA cell loading are specific: superoxide anion generation, but also quite rapid loss of glutathione activity (Fig. 4d) and glutathione peroxidase depletion (Fig. 4e) associated with lipid peroxidation and protein carbonylation, as described above (Fig. 4f, g). All these data summarized in Table 1 characterize classic ferroptosis at high Fe-NTA concentration, i.e. 250 or 500 µM for 48-h incubation. But, in the conditions we currently use with 100 µM Fe-NTA and 24-h incubation, we recorded a low level of cell death.

### Cell death induction by curcumin

Huh-7 cells treated with 25 μM curcumin for 48 h and double-stained with YO-PRO-1 versus PI contained different populations, including viable cells (Y^−^/PI^−^), apoptotic cells (Y^+^/PI^−^ or Y^+^/PI^intermediate^, i.e. slightly positive) and cells undergoing secondary necrosis (Y^+^/PI^+++^). Apoptosis peaked at 25 μM for 24-h incubation, since a higher curcumin concentration (50 μM) favored necrosis (Fig. 5a). It is interesting to note that there is a huge difference between 10 and 25 μM curcumin treatment and also that cumulated cell death (apoptosis + necrosis) reached 60–70% at 25 μM curcumin with 24-h incubation. Clearly, that is not the case with 10 μM curcumin, where overall cell death reached only 7% at 48 h.

As curcumin binds to the endoplasmic reticulum^44^ (Rainey N. et al., publication submitted), it is not surprising that the apoptotic processes are linked to calcium release from the endoplasmic reticulum (Fig. 5c) during the first 4 h of curcumin internalization. This calcium release directly influences mitochondrial behavior, so the mitochondrial membrane potential drop (ΔΨm) (Fig. 5c) together with increased production of superoxide anions (Fig. 5c) is associated with opening of the mitochondrial permeability transition pore. Following this decrease in ΔΨm, there is marked activation of caspase-8 and caspase 3/7, as well as a switch of NAD(P)H and NADH (fluorescent form) to the reduced forms, NAD^+^ and NADP^+^ (which are not fluorescent), thus resulting in a drop in NAD(P)H that parallels the ΔΨm decrease in fluorescence (Fig. 6c). Clearly, if a comparison is made between the apoptosis/necrosis curves and the underlying physiological events, there is a correlation between disruption of mitochondrial homeostasis (i.e. ΔΨm decrease and O_2_^.-^ production) and the early increase of apoptotic events followed by secondary necrosis (Fig. 6b). Curcumin loading is quite rapid and is associated with an immediate increase in red acridine orange fluorescence indicating early induction of autophagic processes (Fig. 6d). The correlation between red acridine orange fluorescence and LC3-II occurrence has been previously described^44^ and suggests that at certain curcumin concentrations the primary events are linked to autophagic induction.

### Iron chelation with curcumin results in a full inhibition of curcumin-induced autophagy and apoptosis

Figure 6 shows that cells treated with curcumin alone undergo autophagy (Fig. 6d), apoptosis (* in Fig. 6a, b), and necrotic death secondary to apoptosis (Fig. 6a), characterized by a progressive loss of viability, a drop in ΔΨm associated with increased superoxide anion production and low activation of caspase-8, caspase-3 and caspase-9 (Fig. 6c). Note that caspase-8 is only secondarily activated as a consequence of lysosomal membrane permeabilization, which is caused by curcumin binding [44] and occurs upstream of ER membrane damage. Then, caspase-8 activated by cathepsin release from the lysosomes induces additional destabilization of the mitochondrial compartment through tBid generation and cytochrome *c* release, which results in activation of caspase-3/-7 and -9.

Fe^2+^chelation completely abolishes curcumin's capacity to induce cell death. The expression of phosphatidyl serine at the cell membrane (late apoptotic and necrotic event) is abolished, as is the ΔΨm drop usually detected as an early event (Fig. 6a). The production of ROS, superoxide anions (Fig. 6b, upper and lower right panels) and hydroperoxides associated with mitochondrial homeostasis disruption is controlled (Fig. 6b). Also, a large panel of metabolic characteristics that are regulated by curcumin-induced cell death are completely inhibited. The mitochondrial membrane potential is high and NAD(P)H fluorescence usually too. Also, caspase-3/7, -8 and -9 are no longer activated, as they were with curcumin treatment alone (Fig. 6c). Moreover, the red acridine orange fluorescence linked to the increasing amount and bigger size of acidic vesicles linked to autophagy enhancement almost falls to control levels (Fig. 6d), whatever the curcumin concentration up to 50 μM. Curcumin-induced autophagy diminishes with increasing curcumin concentration because apoptosis and necrosis occur as curcumin becomes cytotoxic for the cells (Fig. 6d). Below 10 μM, curcumin usually does not promote cell death, but autophagy is engaged. Above 10 μM, the number of acridine orange-positive cells decreases as apoptosis and secondary necrosis occurs.

### Iron chelating activity of curcumin alleviates its tumor-promoting effect

**Fig. 7:**
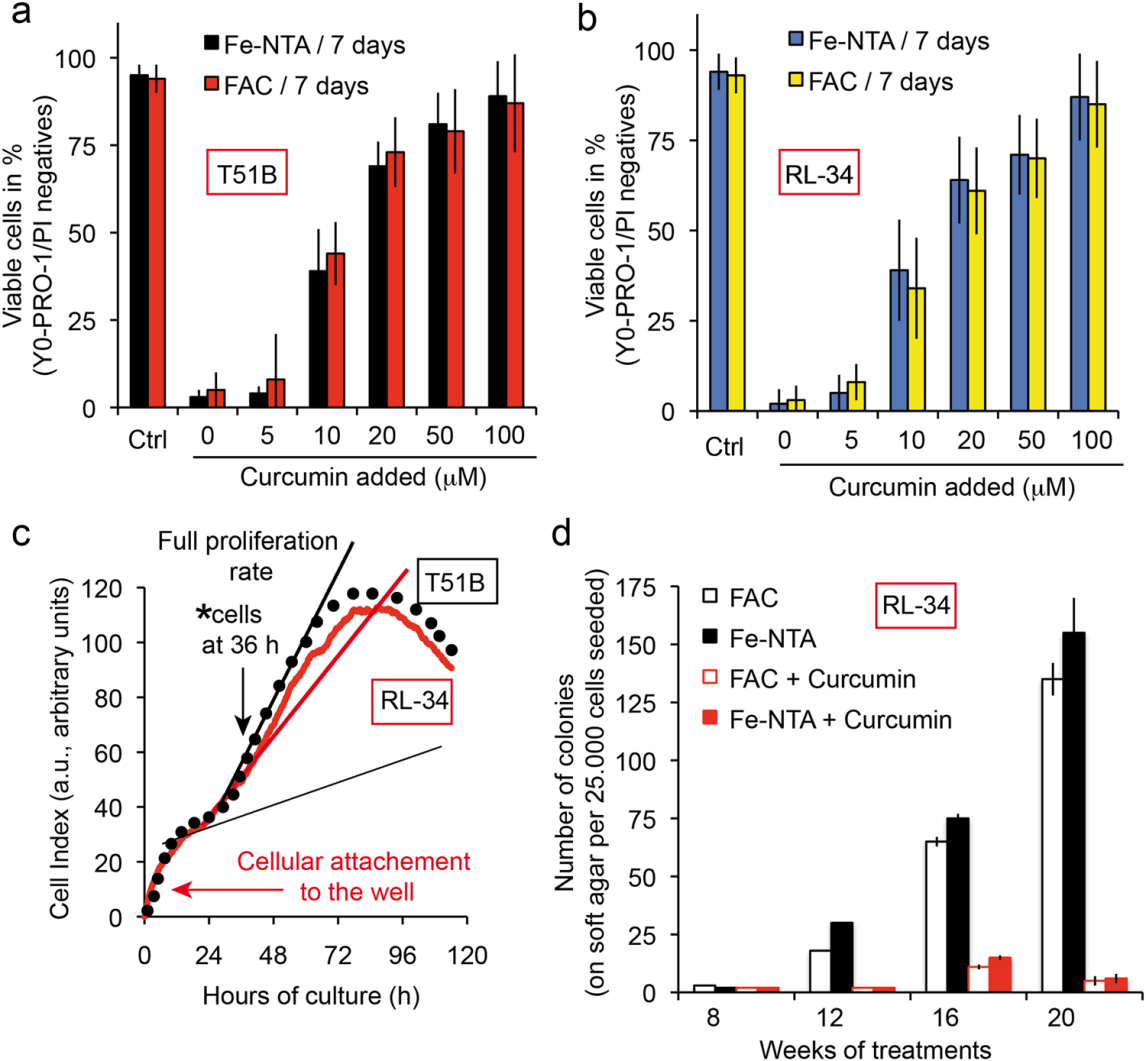
Curcumin chelate with free iron alleviates the tumor-promoting activity of Fe-NTA or FAC. **a** T51B cells are protected against free iron toxicity by curcumin in a concentration-dependent manner. Epithelial T51B cells were treated for 7 days with either 100 µM Fe-NTA or 100 µM FAC ( + 10 µM hydroxyquinoline) and various curcumin concentrations and then viability-tested by cytometry taking into account the dead cells (apoptotic, A and necrotic, N cells) to find the cellular viability [100 − (A + *N*) = viable cells in percentage]. **b** Same as in **a**, but with RL-34 cells. **c** The cells used in the c experiment were taken at 36 h in their full proliferation phase after plating. The situation was tested by impedancemetry using the ACEA Xcelligence system. Arrows (black) indicate the situation of the cells at 36 h and the arrow (in red) early in the curve indicates the initial cell binding to the wells, which in our case took almost 24 h before full proliferation. The curve is drawn taking into account the mean value for 12 different wells. **d** T51B cells plated at 2.10^5^ cells in 60 mm dishes were treated with MNNG (0.5 mg/mL for 24 h) after plating and cultured with either 20 μM curcumin or Fe-NTA 200 μM (or FAC 250 μM with 8-hydroxyquinoline), or both. The culture medium was renewed every 2–3 days with fresh media. T51B cells were trypsinized before they became confluent (day 4–6). The culture continued for up to 20 weeks and colony formation was tested every two weeks, special care being taken when formation became greater at 16, 18, and 20 weeks of culture). The number of soft agar experiments conducted was *n* = 7 and ± SD are indicated. MNNG was added at the start to participate in the initiation of tumorigenesis.

To assess whether curcumin, as a potent iron chelator, can also reduce or prevent the tumor-promoting effect of iron overload, we used T51B (rat liver epithelial cell) and RL-34 (rat liver epithelial diploid cell line). We chose to use the Xcelligence impedancemetry to set up the correct time for the seeding of the cells on soft agar, and decided to take the cells after 36 h when their proliferation peaks (Fig. 7b, indicated by an arrow). This was done for both T51B and RL-34 cell lines. Previous studies show that iron acts as a tumor promoter in T51B cells, with FAC induction associated with a low dose of MNNG generally acting by successive cycles of hepatotoxicity followed by regrowth and regeneration^66^. Acute iron overload was achieved with 250 µM Fe-NTA or with FAC + 8HQ for 7 days in the absence or presence of increasing amounts of curcumin.

The two cell lines exhibited similar behavior and 50 µM curcumin was enough to efficiently counteract iron overload toxicity (Fig. 7b, b) in the case of RL-34. The results (not shown) were very similar with the T51B cells.

Whatever the iron overload protocol, the induction of colonies took over 12 weeks of repeated cycles of cell culture on soft agar, and the number of colonies was quite significant at 20 weeks of culture (over 120 colonies of 25,000 cells) (Fig. 7d). By chelating iron, curcumin controls colony development and after 20 weeks of culture of both cell lines the number of colonies did not exceed five (Fig. 7d).

## Discussion

### Curcumin-iron interactions

Cyclic voltammetry experiments show that curcumin binds metal ions and especially Fe^2+,^^67^. The iron chelation activity of curcumin is related to its chemical structure via the β-diketone group, a known bidentate chelator of Fe^2+^, similar to the group found in simple analogous complexes of Fe(III) and acetylacetonate. Based on the formation constant of Fe(III)-curcumin of 10^22^ M^−1,^^67^, it has been calculated that the pFe^2+^ (‘‘pM’’) of curcumin is 16.6 (at pH 7.4 for 10 μM curcumin and 1 μM Fe^2+,^^68^). This curcumin pM is not as high as the pM of other iron chelators already used in iron-overload treatment, such as deferiprone (pM = 20) and deferoxamine (pM = 26)^68^. Curcumin pM compares favorably with the pM of the iron chelator nitrilotriacetic acid (NTA), which we use on the nickel column for extraction of curcumin and many other iron chelators (Fig. 1) and fits well with other iron chelating activity detected in vitro^68^. The methods used for loading iron into cells yield endogenous iron contents ranging from 7.2 ± 1.9 μg iron/g of cells to 28 ± 10 μg/g of cells, for 48-h incubation with 100 μM Fe-NTA (Fig. 3a). These results are not altered by the deleterious effect of iron, as its toxicity becomes significant only above 100 µM and for longer time periods. Iron toxicity is associated with lipid peroxidation and protein carbonylation (Fig. 4d, e, g) and a decrease in intracellular protection against ROS-like glutathione activity (Fig. 4b, c). Iron accumulation at higher Fe-NTA concentrations for 48 h leads to intracellular accumulation of 60.5 ± 13.2 and 108.5 ± 19.6 μg/g for Fe-NTA 250 and 500 μM, respectively (Table 1). These conditions of loading go beyond the capacity of the endogenous antioxidant defense system, with clear depletion of glutathione activity, which is overwhelmed by massive generation of ROS from cellular Fe^2+^ engaged in Fenton reactions/Haber–Weiss reactions^2,3^.

Although curcumin clearly binds iron, there is no significant reduction in iron uptake by cells (Fig. 3a), though there may be a discrepancy due to transient interaction between curcumin and the plasma membrane during primary penetration, when it could interact with the iron regulation system. So, our data show that curcumin binding to iron does not influence intracellular iron levels in response to Fe-NTA treatment. We therefore investigated whether curcumin chelation of iron is still hermetic behavior or whether its activity is altered.

### Curcumin cellular effects

We investigated the Huh7 profile response to curcumin as previously^44^ and confirmed that curcumin affects the ER via calcium release taken up by mitochondria via the mitochondrial calcium uniporter, resulting in a drop in ΔΨm and superoxide anion production, leading ultimately to opening of the mitochondrial permeability transition pore (Fig. 5c). These results suggest the further involvement of cathepsins and caspase-8 activation leading to cytochrome c release and apoptosis, as previously described^44^. This does not exclude early involvement of lysosomes, as it is clear that curcumin in the µM range induces autophagic processes (Fig. 5d)^44^. This may explain published observations on the acceleration of neoplastic transformation when 10–20 μM curcumin is added to iron^29,69^. It is possible that 10–20 µM curcumin is more than enough to chelate all iron and that the remaining iron-free curcumin induces autophagy, enhancing neoplastic transformation^44,47^. Indeed, in neoplastic cells, autophagy is a mechanism for coping with intracellular and environmental stress, thus favoring tumor progression^70^.

### Curcumin-iron chelates abolish both curcumin effects and iron cytotoxicity

When chelated with iron, curcumin loses its capacity to induce toxicity and cell death (Fig. 6), which is in line with previous reports showing that iron attenuates curcumin's cytotoxic effects in squamous cell carcinoma^71^. Chelation abolishes late stage signals like outer exposure of phosphatidyl serine (Fig. 6a), but also early production of both hydrogen peroxide and superoxide anions from the mitochondrial compartment (Fig. 6b). These results fit with other inhibition linked to cell death, like the drop in mitochondrial potential, reduction of NAD(P)H, and activation of caspases 3/9/8 (Fig. 6c). These results contrast with the ongoing hypothesis that combination of ions and ligands would produce a synergistic effect on cells^72–74^. A possible explanation may come from the fact that the keto-enol function and related hydrogen that is locked with metals are unable to produce oxyradicals unless there is complete dissociation. According to these data, the keto-enol function is also likely to drive the curcumin-induced autophagic process (Fig. 6d). Another hypothesis to consider would be the modification of the size and steric hindrance of the curcumin chelate compared to free curcumin, which would open up a new set of possible mechanisms yet to be studied.

We used the soft agar tumorigenesis model to investigate for longer periods how RL-34 cells respond to iron-induced stress (with an MNNG starter) and curcumin treatment. As FAC or Fe-NTA treatment (250 µM) produced large numbers of colonies, 20 µM curcumin is enough to abolish that growth (Fig. 7d). Messner and colleagues recently found this same response for 20 µM curcumin, but observed tumor promotion for 10 µM curcumin in the same settings^69^. We hypothesize that 10 µM curcumin is already in excess compared to free iron, especially if curcumin can cycle the iron to transferrin and maintain it in the redox inactive state^52^. That excess is not enough to induce apoptosis and control tumorigenesis like 20 µM does (Fig. 7d), but is enough to stimulate autophagic processes and therefore boost tumorigenesis.

In a clinical cancer setting, we would need to address a specific curcumin payload, either partially chelating iron to reduce this tumor-promoting signal or increasing the payload enough to produce apoptosis and not boost autophagy.

Iron-overload diseases are not subject to this limitation, and while iron chelation is sought, an extra autophagic signal could be valuable for patients at the tissue level, as could the anti-oxidative properties provided by low concentrations of curcumin.

## Materials and methods

### Chemicals and reagents

Calcein-AM, curcumin, deferoxamine, 8-hydroxyquinoline (8-HQ), ferric ammonium citrate (FAC), propidium iodide (PI), *N*-acetylcysteine (NAC) were from Sigma-Aldrich Chemical Co. (St. Louis, MO, USA). Calcein-AM, 2,7-dichlorodihydrofluorescein diacetate (DCFH-DA), 3,3′- dihexyloxacarbocyanine iodide [DiOC_6_(3)] and *N*-[4-[6-[(acetyloxy) methoxy]-2,7-dichloro-3-oxo-3H-xanthen-9-yl]-2-[2-[2-[*bis*[2[(acetyloxy) methoxy]-2-oxyethyl] amino]-5-methyl-phenoxy] ethoxy]phenyl- *N*-[2-[(acetyloxy) methoxy]-2-oxyethyl]-(acetyloxy) methyl ester (Fluo-4/AM) were from Molecular Probes (Invitrogen, Eugene, OR, USA). Agarose was from Lonza (Walkersville, MD, USA. 8-Hydroxyquinoline was given together with FAC to enhance its internalization.

### Cells

Human hepatoma-derived Huh-7 cells were from the RIKEN BioResource Center, Tsukuba, Japan and were grown in the presence of 5% CO_2_ with Dulbecco’s modified Eagle’s medium (DMEM) containing high glucose (25 mM Sigma-Aldrich, St. Louis, MI) with 10% fetal bovine serum (FBS, Hyclone, Logan, UT) completed with 1% penicillin-streptomycin, HEPES NaOH 1 mM, Na-pyruvate 1 mM and 1% non-essential amino acids (MEAM, GIBCO). T51B cells (rat epithelial cell lines, non-neoplastic) are a model for the study of tumor promotion in vitro. They can be transformed to grow in soft agar by treatment with small amounts of carcinogens and tumor promoters^75^. RL-34 cells (rat liver epithelial-like cells, non-neoplastic from JCrB Bank, Japan) were maintained in the same medium as T51B cells.

### Cell culture, iron overload and neoplastic transformation

The neoplastic transformation of T51B and RL-34 cells was done as described by Messner et al.^69^ and the colony formed on soft agar counted as a transformation index^75^. We followed exactly the previous transformation protocol with a single 24-h treatment with 0.5 μg/mL *N*-methyl-*N*′-nitrosoguanidine (MNNG) followed by continuous culture for weeks either with ferric ammonium citrate (FAC) plus 8-HQ or 250 μM Fe-NTA with or without 25 μM curcumin.

### Intracellular iron dosage

Intracellular iron contents of Huh7 cells given in micrograms of iron per gram of cells were determined after treatment with 100 µM Fe-NTA and/or 100 µM DFO for 24 h. Cell cultures (150 cm^2^ at 80% confluence) were trypsinized and collected with lysate buffer (trichloroacetic acid 0.1 g/L, hydrochloric acid 0.774 M, mercaptoacetic acid 30 mM) to give 100 mg of cells for 400 µL buffer. Lysate was heated to 65 °C overnight and centrifuged. Supernatants were then analyzed by flame emission spectrometry (Institut Claude Bernard, ICB, Paris) and compared to standards to determine iron content.

### Iron affinity resin

Fe-NTA-agarose was constructed from commercially available nickel NTA agarose (Qiagen, S.A., France). The Ni-NTA agarose was stripped with EDTA and recharged with iron as described^64^. Binding and pull-down experiments were performed in 50% ethanol and assumed 100% replacement of Ni sites with Fe^2+^ (as guaranteed by the manufacturer). For the binding experiments, the indicated amounts of curcumin were incubated with or without Fe-NTA-agarose in 50% ethanol at room temperature (roughly 25 nmol metal binding sites in 0.5 mL total volume). After 10 min, the resin was removed by centrifugation, and aliquots of the supernatants were diluted as needed (10-fold) to determine the curcumin remaining in solution (absorbance at 435 nm). Affinity estimations were based on the method of Scatchard, assuming free curcumin equal to the amount remaining in solution in the presence of Fe-NTA-agarose, and bound curcumin equal to the amount of curcumin added minus the free curcumin measured at each concentration shown. The experiments were conducted exactly as described by Messner et al.^52^.

### Determination of lipid peroxidation and protein carbonylation

We used a lipid peroxidation assay kit (Abcam) to detect malondialdehyde (MDA) in samples. The free MDA generated during lipid peroxidation refers to the oxidative degradation of lipids reacting with thiobarbituric acid (TBA) to generate an MDA-TBA adduct. The absorbance of MDA-TBA adduct was measured at 532 nm for a sensitivity as low as 1 nmol/well. For the calculation, we determined the MDA concentration in standards and samples from their absorbance as described in the protocol of the lipid peroxidation assay kit from Abcam (ab118970). Protein carbonylation was assayed in Huh-7 cell lysates using Cayman’s Protein Carbonyl Fluorometric Assay Kit.

### Microspectrofluorimetry

The UV-visible confocal laser microspectrofluorometer prototype was built around a Zeiss UMSP80 UV epifluorescence microscope (Carl Zeiss, Inc., Oberkochen, Germany), optically coupled by UV reflecting mirrors to a Jobin-Yvon HR640 spectrograph (ISA, Longjumeau, France)^76^. The 351-nm UV line of an argon laser (model 2025; Spectra-Physics, Mountain View, CA) was used for either drug or fluorochrome excitation. The diameter of the laser beam is first enhanced through a double-lens beam expander in order to cover the entire numerical aperture of the microscope's optics. The laser beam is then deflected by the epi-illumination system (dichroic mirror or semireflecting glass) and focused on the sample through the microscope objective (X63 Zeiss Neofluar water-immersion objective; numerical aperture = 1.2) on a circular spot 0.5 µm in diameter. The excitation power is reduced to less than 0.1 μW by neutral optical density filters. The objective was immersed in the culture medium, and a circular area 0.8 μm in diameter was selected at the sample level, by interposing a field diaphragm on the emission pathway of the microscope, to selectively collect the fluorescence signal from the nucleus or a specific cytoplasmic area. Confocal conditions are met when the image of this analysis field diaphragm through the microscope objective perfectly coincides with the focus of the laser beam on the sample.

Under these conditions, the experimental spatial resolution, measured on calibrated latex beads (2, 0.6, and 0.16 µm in diameter) labeled with the fluorescent probe fluorescein, is 0.5 µm for the directions X, Y, and Z. Finally, the fluorescence spectra were recorded after spectrograph dispersion, in the 380–630 nm region on a 1024 diode-intensified optical multichannel analyzer (Princeton Instruments, Inc., Princeton, NJ) with a resolution of 0.25 nm/diode. Each fluorescence emission spectrum was collected from 1 to 10 s. Data were stored and processed on an 80286 IBM PS/2 microcomputer using the Jobin-Yvon "Enhanced Prism" software. It should be noted that, in order to avoid any possible fluorescence from a plastic or glass support during analysis with near-UV excitation, cells were grown on quartz plates that were then placed on the microscope stage in 50-mm thermostated Petri dishes, filled with 5 mL of phosphate buffered saline (PBS). A uranyl glass bar was used as a fluorescence standard to control laser power and instrumental response and to enable quantitative comparison between spectra recorded on different days. Sample heating, photobleaching, and photo damage were assessed empirically and found to be negligible under our experimental conditions. In particular, cells always remained viable after repeated fluorescence determinations, as checked by phase-contrast microscopy.

### Determination of cellular viability, mitochondrial membrane potential (ΔΨm), reactive oxygen species and cytosolic Ca^2+^ levels

A density of 2 × 10^6^ Huh-7 cells on six-well plates were maintained with 25 μM of curcumin for a given period of time ranging from 0 to 48 h depending on the experiments. After treatment, cells were trypsinized, harvested, washed and then resuspended together with their supernatant in PBS. 3,3′-Dihexyloxacarbocyanineiodide [DiOC_6_(3)] was added to a final concentration of 40 nM for ΔΨm determination, 2′,7′-dichlorodihydrofluorescein diacetate (DCFH-DA) to 5 μM for H_2_O_2_, and MitoSOX to 1 μM for superoxide anion. Most of the time double staining was done in order to assay simultaneously cellular viability, with propidium iodide (PI: stock solution; 1 mg mL^−1^) for DiOC_6_(3), DCFH-DA and Fluo4-AM and with 2 μg/mL TO-PRO-3 iodide (stock solution; 1 mg mL^−1^) for MitoSOX. Supplemental double staining was used for the distinction between viable, apoptotic and necrotic cells with YO-PRO-1 / PI (Molecular Probes) in parallel with annexin-V/PI staining done with annexin-V-FITC when needed (Immunotech, Beckman-Coulter). All samples were analyzed by flow cytometry as previously described^77,78^ on a FACS Calibur 4C.

### Use of monochlorobimane for detection of intracellular GSH activity

The Huh-7 cell content of GSH was determined using 50 μM monochlorobimane (mBCl) in 20-min incubation at room temperature in the dark^78^. Monochlorobimane (Molecular Probes, Eugene, OR) was dissolved in 100% ethanol to a stock concentration of 40 mM and stored at −20 °C. Special precautions were used to minimize the exposure of mBCl to ambient light. To assess mBCl for detection of intracellular GSH, Huh-7 cells were also incubated with *N*-ethylmaleimide (NEM; Sigma) as a control, a GSH depleting agent, which has been used previously to establish the specificity of mBCl for detection of GSH^79^. N-ethylmaleimide was prepared as a stock solution in 100% ethanol and was added to suspensions of cells to a final concentration of 100 mM for 10 min at room temperature prior to addition of mBCl. Monochlorobimane fluorescence was assessed using a UV laser with excitation wavelength at 360 nm and an emission at 585 ± 42 nm (Fl-2) set on a FACS Aria (Becton-Dickinson, USA).

### Glutathione peroxidase assay

For glutathione peroxidase determination, we used the Glutathione Peroxidase Assay Kit (Colorimetric) from abcam and followed the manufacturer's instruction. The data are expressed in enzymatic units per mg protein.

### Calcein quenching experiments

In order to measure calcein quenching, cells were loaded with 0.25 μg/mL calcein-1AM in serum-free media for 30 min at 37 °C, rinsed, and then treated (in triplicate) as specified in complete culture media for 2 h. The cells were rinsed three times with PBS, and green fluorescence was measured by flow cytometry at 525 ± 10 nM (FL-1).

### Caspase activation, fluorimetric assays

Isolated Huh-7 cells were washed and suspended in calcium-free buffer solution (140 mM NaCl, 1.13 mM MgCl_2_, 4.7 mM KCl, 10 mM glucose, 0.1 M EDTA, and 10 mM HEPES, pH 7.2). Cells untreated or treated with 20 µM curcumin for 24 h were then loaded at room temperature for 30 min with fluorescent indicator-linked substrates presented as kits–Vybrant™ FAM Caspase-3 and -7 Assay Kit - CaspGLOW™ Fluorescein Active Caspase-9 Staining Kit and the Vybrant FAM caspase-8 assay kit (V35119), all from Molecular Probes, and analyzed by flow cytometry. The percentage of cells (%) that are viable or in early apoptosis (PI negative or with a slight increase in PI) with caspase activity greater than that of the control was taken into consideration, whereas the cells that were totally permeable to PI were excluded, as were the control cells (negative for PI and almost negative for caspase activity).

## Supplementary information

data set 1
