# Supplementary material for: Iron chelation by curcumin suppresses both curcumin-induced autophagy and cell death together with iron overload neoplastic transformation
Source: Cell Death Discov. 2019 Dec 9;5:150. doi: 10.1038/s41420-019-0234-y (PMC6901436; doi:10.1038/s41420-019-0234-y)
Supplement: Supplementary file 1 — data set 1 [file 41420_2019_234_MOESM1_ESM.docx]

**Detailled Attribution of Autorship**

Conception and design: PXP; Data acquisition and analysis: PXP, NER, AM, ASA. Microspectrofluorimetry was carried out by PXP and FS. Confocal microscopy was done by AS, PXP. NER and ASA did the Western blots, GN contributed to the initiation of this work and helped us with the Fe-NTA loading work. All authors contributed to the review and revision of the manuscript. All authors read and approved the final manuscript.
